# A five-miRNA signature with prognostic and predictive value for *MGMT* promoter-methylated glioblastoma patients

**DOI:** 10.18632/oncotarget.4978

**Published:** 2015-07-22

**Authors:** Wen Cheng, Xiufang Ren, Jinquan Cai, Chuanbao Zhang, Mingyang Li, Kuanyu Wang, Yang Liu, Sheng Han, Anhua Wu

**Affiliations:** ^1^ Department of Neurosurgery, The First Hospital of China Medical University, Shenyang, China; ^2^ Department of Pathology, Shengjing Hospital of China Medical University, Shenyang, China; ^3^ Department of Neurosurgery, The Second Affiliated Hospital of Harbin Medical University, Harbin, China; ^4^ Beijing Neurosurgical Institute, Beijing, China; ^5^ Department of Neurosurgery, Beijing Tiantan Hospital, Capital Medical University, Beijing, China; ^6^ Department of Neurosurgery, The First Affiliated Hospital of Dalian Medical University, Dalian, China

**Keywords:** glioblastoma, MGMT promoter methylation, miRNA, prognosis, predictive model

## Abstract

Although *O(6)-methylguanine DNA methyltransferase* (*MGMT*) promoter methylation status is an important marker for glioblastoma multiforme (GBM), there is considerable variability in the clinical outcome of patients with similar methylation profiles. The present study aimed to refine the prognostic and predictive value of *MGMT* promoter status in GBM by identifying a micro (mi)RNA risk signature. Data from The Cancer Genome Atlas was used for this study, with *MGMT* promoter-methylated samples randomly divided into training and internal validation sets. Data from The Chinese Glioma Genome Atlas was used for independent validation. A five miRNA-based risk signature was established for *MGMT* promoter-methylated GBM to distinguish cases as high- or low-risk with distinct prognoses, which was confirmed using internal and external validation sets. Importantly, the prognostic value of the signature was significant in different cohorts stratified by clinicopathologic factors and alkylating chemotherapy, and a multivariate Cox analysis found it to be an independent prognostic marker along with age and chemotherapy. Based on these three factors, we developed a quantitative model with greater accuracy for predicting the 1-year survival of patients with *MGMT* promoter-methylated GBM. These results indicate that the five-miRNA signature is an independent risk predictor for GBM with *MGMT* promoter methylation and can be used to identify patients at high risk of unfavorable outcome and resistant to alkylating chemotherapy, underscoring its potential for personalized GBM management.

## INTRODUCTION

Glioblastoma multiforme (GBM) is the most common and deadly type of malignant tumor in the central nervous system. The median survival time of GBM is 14 months even after standard treatment consisting maximal surgical resection followed by adjuvant chemotherapy and radiotherapy [1]. However, the survival time varies widely from < 3 months to > 3 years following diagnosis [1], underscoring the limitations of current clinicopathologic markers and grading systems in predicting patient outcome. Recent studies have identified many markers for GBM; one of the most reliable of them is the methylation status of the *O(6)-methylguanine DNA methyltransferase* (*MGMT*) gene promoter. Epigenetic silencing of the MGMT gene by promoter methylation is associated with longer survival time and increased sensitivity to chemotherapeutic alkylating agents in GBM patients [2, 3]. However, patients with equivalent *MGMT* promoter methylation status have variable prognoses and responses to treatment [4], suggesting that other factors are equally important in determining clinical outcome.

Micro (mi)RNAs are small noncoding RNAs that post-transcriptionally regulate target gene expression and thus function as tumor suppressors or oncogenes. Recent reports suggest that miRNA expression-based clustering could be a more accurate means for tumor classification and prediction than clustering based on mRNA expression profiles [5, 6]. Several miRNAs have been correlated with glioma progression and prognosis [7-11]; however, despite the reported link between miRNA expression and the prognosis of GBM specifically [9-11], there have been no studies exploring the prognostic role of miRNAs with respect to *MGMT* promoter methylation status. In the present study, we performed miRNA expression profiling in a cohort of 150 primary GBM cases with MGMT promoter methylation. A survival analysis revealed a five miRNA-based risk signature specific for *MGMT* promoter-methylated GBM, which was validated using an independent set. The five-miRNA signature identified patients who had a high risk of unfavorable outcome and were resistant to alkylating chemotherapy, thereby refining the predictive value of *MGMT* promoter methylation.

## RESULTS

### Identification of a five miRNA-based risk signature in the training set

A total of 150 primary GBM with *MGMT* promoter methylation and 10 non-cancerous brain tissue samples were included in the comparison. A total of 171 miRNAs were differentially expressed in these samples (false discovery rate, FDR < 0.01) (Supplementary Data 1). We randomly assigned the 150 samples to training or validation set, which did not differ in terms of clinicopathologic features (Table 1). A univariate Cox regression analysis was used to evaluate the prognostic value of the 171 miRNAs in training set. Five (miR-222, -145, -20a, -132, and -129) were significantly associated with overall survival (OS) (*P* < 0.05; Table S1 and Supplementary Data 2). These were of two types: i.e., risky or protective. MiR-222, -132, and -129 were defined as risky miRNAs with a hazard ratio (HR) > 1 for death, whereas miR-145 and -20a were defined as protective with HR < 1. The expression levels of the five miRNAs were compared in normal brain and *MGMT* promoter-methylated GBM tissue (Supplementary Figure S1).

**Table 1:** Clinical and molecular pathology features between training and validation sets

Characteristic	Training set (N=75)	Validation set (N=75)	P-value
**Gender**			1.0000*
Male	38 (51%)	385 (51%)	
Female	37 (49%)	37 (49%)	
**Age, years**			0.1218†
Mean (range)	58.5 (10.9-85.2)	56.5 (19-85.6)	
**Age, per 20 years**			0.3637*
<30	3	3	
30-49	16	17	
50-69	34	42	
≥70	22	13	
**KPS**			0.8412*
≥80	37 (67%)	42 (70%)	
<80	18 (33%)	18 (30%)	
**IDH1 mutation**			0.6091*
Mutation	7 (11%)	10 (15%)	
Wild type	55 (89%)	57 (85%)	
**Radiation**			1.0000*
Yes	71	71	
No	4	4	
**Chemotherapy**			0.5408*
Yes	62	58	
No	13	17	

**Data are mean n (%) or otherwise stated.**

* χ^2^ test or Fisher's exact test, †Student's t-test.

**Figure 1:**
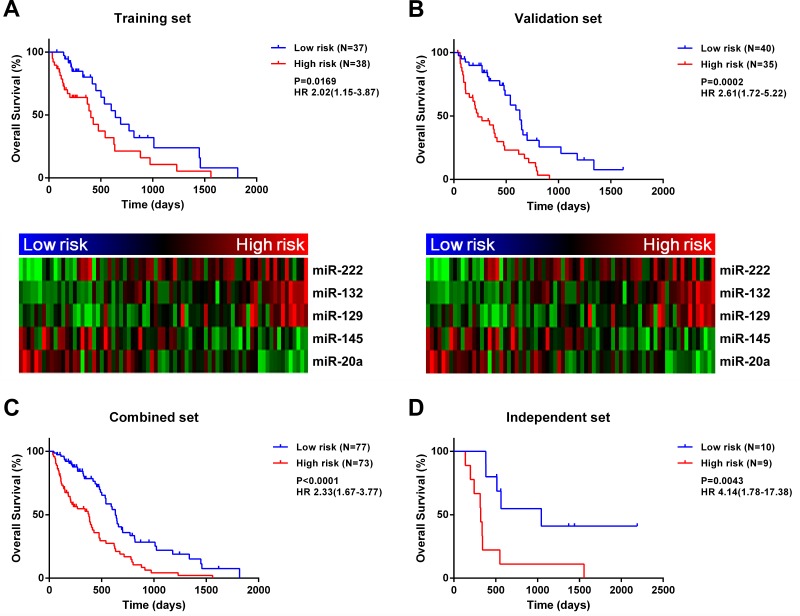
A five-miRNA signature is closely associated with *MGMT* promoter-methylated GBM prognosis A five-miRNA signature divides patients into two groups with significantly different prognosis in training **A.**, validation **B.**, combined **C.**, and independent **D.** datasets.

We developed a risk signature for each patient based on the expression of the five miRNAs [12, 13] as follows: risk value = (0.4715 × expression level of miR-222) + (0.5279 × expression level of miR-132) + (0.3327 × expression level of miR-129) − (0.5432 × expression level of miR-145) − (0.4820 × expression level of miR-20a). Patients in the training set were divided into high- and low-risk groups based on the median risk value (0.0178) used as the cutoff. High-risk patients had significantly shorter survival time than those in the low-risk group (median OS = 404 *vs*. 641 days; HR = 2.02, 95% confidence interval, CI: 1.15-3.87, *P* = 0.0169; Figure 1A). Meanwhile, risky and protective miRNAs exhibited distinct expression patterns corresponding to the risk value (Figure 1A); that is, high-risk patients expressed higher levels of risky miRNAs (miR-222, -132, and -129), while low-risk patients expressed higher levels of protective miRNAs (miR-145 and -20a).

### Validation of the five-miRNA signature for predicting survival in validation, combined, and independent sets

The five miRNA-based signature was determined for each sample in the validation set. Samples were classified into high- (*n* = 35) or low-risk (*n* = 40) groups with the same cutoff value (risk value = 0.0178) that was used for the training set. As expected, OS was reduced for high-risk patients in the validation set (median OS = 230 *vs*. 632 days; HR = 2.61, 95% CI: 1.72-5.22, *P* = 0.0002; Figure 1B). Meanwhile, risky miRNAs were overexpressed in the high-risk group, whereas protective miRNAs were overexpressed in the low-risk group. When patients from the training and validation sets were combined, the five-miRNA signature also conferred unfavorable prognosis for high-risk patients (median OS = 378 *vs*. 632 days; HR = 2.33, 95% CI: 1.67-3.77, *P* < 0.0001; Figure 1C). To determine whether the prognostic value of the five miRNA-based signature was applicable to different populations, we analyzed an independent validation set of 19 patients with *MGMT* promoter methylation from the Chinese Glioma Genome Atlas (CGGA) database. Patients were divided into high- (*n* = 9) or low-risk (*n* = 10) groups using the same cutoff value as used for the training set (0.0178). A Kaplan-Meier analysis indicated that OS was significantly shorter in high- than in low-risk patients (median OS = 320 *vs*. 1045 days; HR = 4.14, 95% CI: 1.78-17.38, *P* = 0.0043; Figure 1D). Similarly, progression-free survival (PFS) was reduced for high-risk patients in the independent set (median PFS = 131 *vs*. 664 days; HR = 3.39, 95% CI: 1.44-12.91, *P* = 0.0113; Figure S2).

When this formula was applied to GBM patients without *MGMT* promoter methylation, there was no difference in survival time between high- and low-risk groups irrespective of whether the cutoff value was the same as the one used in the previous analyses or was the median risk score for this group of patients (Supplementary Figure S3A, B).

The expression profiles of cases in the combined set who survived > 1 year and ≤ 1 year were compared with the Student's t test. The expression of miR-222, -145, -20a, and -132 differed significantly (Figure S4); however, not all miRNAs were differentially expressed between patients with long and short survival time, whereas the five-miRNA signature showed greater difference and significance between these two groups, confirming its reliability as a prognostic marker.

### Application of the five-miRNA signature in cohorts stratified by clinicopathologic factors

**Figure 2:**
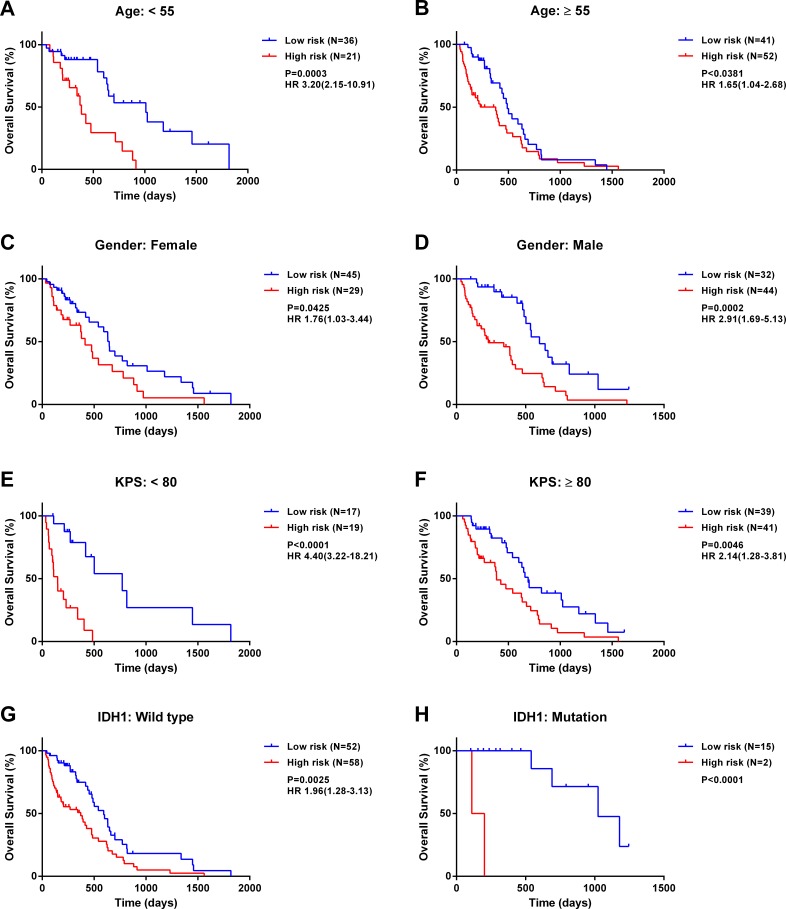
The five-miRNA signature has prognostic significance in different cohorts Kaplan-Meier survival analysis of 150 GBM patients with *MGMT* promoter methylation based on the five-miRNA signature and stratified by the following clinicopathologic risk factors: age **A.**, **B.**, gender **C.**, **D.**, Karnofsky Performance Score **E.**, **F.**, and *isocitrate dehydrogenase 1* mutation status **G.**, **H.**.

We first stratified patients based on several clinicopathologic factors, including age, gender, Karnofsky Performance Score, and *isocitrate dehydrogenase* (*IDH*)*1* mutation status. In all cohorts, high-risk patients had significantly shorter survival than low-risk patients (log-rank test; Figure 2). These results indicate that the five-miRNA-based classification accurately identified patients with poor prognosis irrespective of clinicopathologic risk factors (Figure 2A-2H). Furthermore, an IDH1 mutation was detected in 17 samples of which two were classified as high-risk as compared to 15 that belonged to the low-risk group, suggesting that the signature selects for a GBM subtype with known differences in survival.

### Assessment of the five-miRNA signature in relation to alkylating chemotherapy

Previous studies have demonstrated that *MGMT* promoter methylation indicates increased sensitivity to alkylating chemotherapy for GBM patients [2, 3]. Among cases with *MGMT* promoter methylation, 120 received the recommended chemotherapy, which was either temozolomide (TMZ) (*n* = 113) or other alkylating agents (*n* = 7) [14], whereas 30 patients received no chemotherapy.

**Figure 3:**
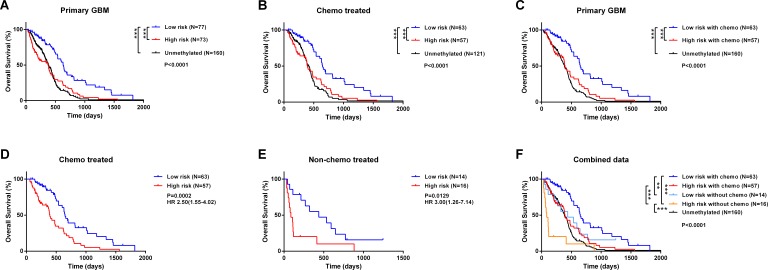
Comparison of prognosis between unmethylated GBM samples and high- and low-risk GBM patients with *MGMT* promoter methylation **A.**-**C.** Survival among GBM patients in different groups stratified by *MGMT* promoter methylation status and the five-miRNA signature. **D.**-**F.** Kaplan-Meier survival curves based on alkylating chemotherapy and five-miRNA signature. *P < 0.05, **P < 0.01, ***P < 0.001.

We compared the prognosis of GBM without *MGMT* promoter methylation with that of patients classified a high- or low-risk. Low-risk patients survived longer than either high-risk or unmethylated patients, who had similar survival times despite differences in *MGMT* promoter methylation status (Figure 3A). This trend was validated in an independent set (Figure S5). For treatment with alkylating agents, the five-miRNA classifier was able to identify a group of high-risk patients with *MGMT* promoter methylation, whose survival time was similar to that of unmethylated patients (Figure 3B). High-risk patients-even those treated with alkylating agents-did not have a survival advantage over the overall unmethylated patients (Figure 3C).

Treatment with alkylating agents can prolong the survival time of *MGMT* promoter-methylated GBM [15-17]. We therefore carried out survival analysis for chemotherapy-treated and untreated patients. High-risk patients had reduced survival time as compared to those at low-risk in both treated and untreated groups (Figure 3D, 3E), implying that the five-miRNA signature can predict prognosis independently of alkylating chemotherapy. We then combined the five-miRNA signature and chemotherapy for survival analysis, which revealed significant differences among subgroups (Figure 3F). Low-risk patients who received chemotherapy had the best prognosis, whereas high-risk patients who did not receive chemotherapy had the worst prognosis even compared to unmethylated patients. There was no difference between unmethylated patients, chemotherapy-treated high-risk patients, and low-risk patients who did not receive alkylating treatment.

Among patients who received alkylating treatment, those with OS > 1 year or ≤ 1 year were defined as chemotherapy responders and non-responders, respectively. The five-miRNA signature and constituent miRNAs were compared between these two groups. Only two individual miRNAs differed significantly between responders and non-responders; however, the five-miRNA signature differed significantly (Figure S6), indicating that combining multiple factors in the evaluation yields more robust and stable results.

### The five-miRNA signature is an independent prognostic factor for GBM with MGMT promoter methylation

In the combined set, a univariate Cox regression analysis revealed that the five-miRNA signature was significantly associated with OS. Multivariate Cox regression analysis provided further evidence for the five-miRNA signature as an independent prognostic factor (HR = 2.1752, *P* = 0.0009; Table 2). Similarly, the signature was validated as an independent prognostic factor using an independent set (HR = 6.4662, *P* = 0.0090; Table S2). These results indicate that the five-miRNA risk signature can independently predict clinical outcome for *MGMT* promoter-methylated GBM cases.

**Table 2:** Cox hazard regression analysis of clinicopathologic factors and the five-miRNA risk signature for survival in the combined set

Variable	Univariate Cox		Multivariate Cox	
	P-value	HR	P-value	HR
**Age**				
(Per 20 years)	<0.0001	2.0169	0.0018	1.8209
**Gender**				
(Female vs. Male)	0.0792	0.6938		
**KPS**				
(≥80 vs. <80)	0.0569	0.6226		
**IDH1 mutation**				
(Mutation vs. Wild type)	0.0164	0.3594	0.5475	0.7522
**Radiotherapy**				
(Treated vs. Untreated)	0.0002	0.2451	0.2634	0.6121
**Chemotherapy**				
(Treated vs. Untreated)	0.0007	0.4519	0.0133	0.4818
**Five-miRNA signature**				
(High vs. Low risk)	**<0.0001**	**2.3887**	**0.0007**	**2.2117**

### Quantitative model for predicting patient prognosis

**Figure 4:**
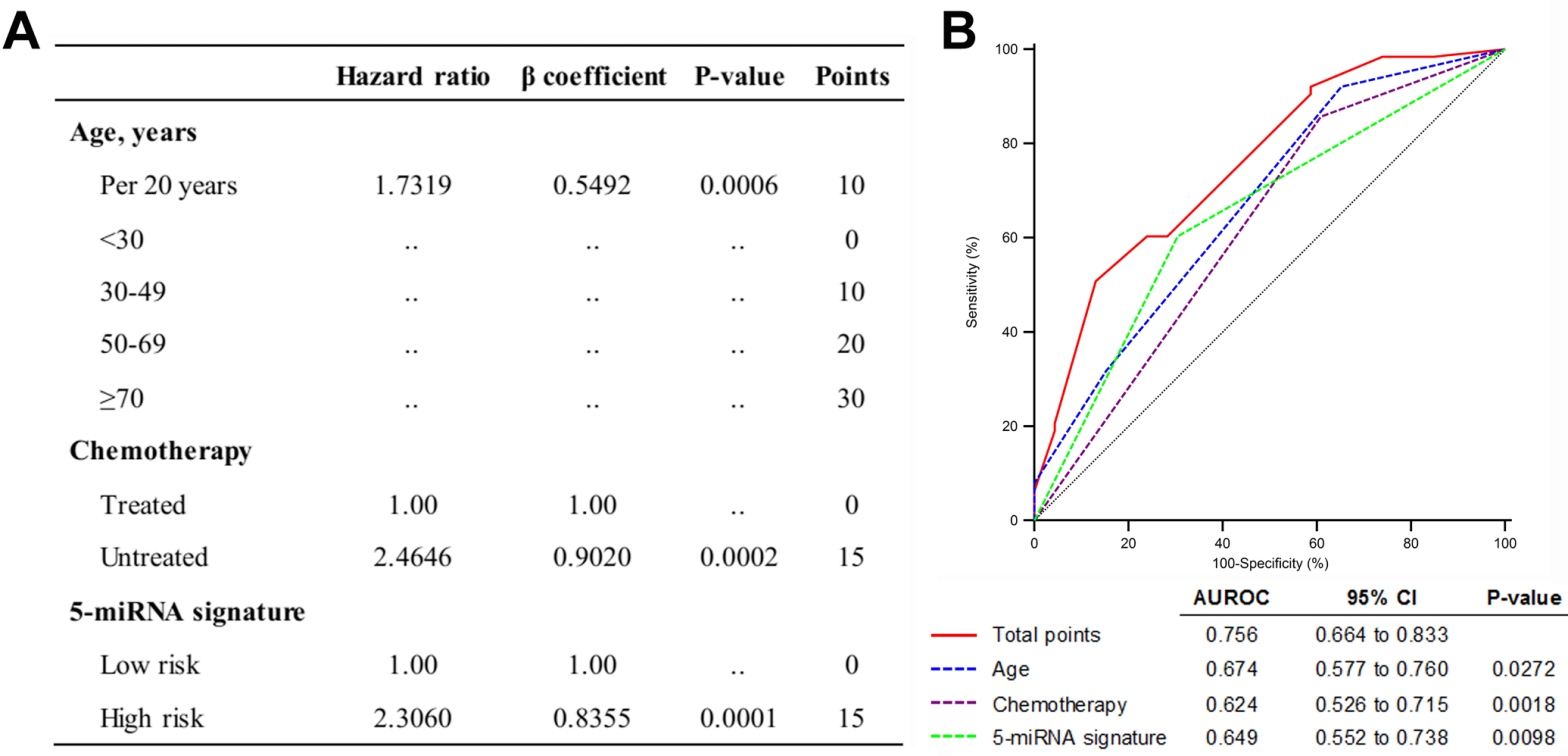
Quantitative model based on three independent prognostic factors **A.** Predictive risk model for GBM patients with *MGMT* promoter methylation. **B.** Comparison of sensitivity and specificity for predicting 1-year survival using the risk model, which includes age, alkylating chemotherapy, and the five-miRNA signature in a combined set of 150 GBM samples with *MGMT* promoter methylation.

Based on the results of the multivariate Cox analysis in the combined set, we developed a predictive model that combined three independent prognostic factors: age, chemotherapy and the five-miRNA signature. A multivariate Cox proportional hazards model was used to estimate the regression coefficient, P value, and HR for each of the three factors. Regression coefficients for chemotherapy and the five-miRNA signature were divided by the coefficient corresponding to a 20-year increase in age, and the value was rounded to the nearest integer to generate a risk point [18] (Figure 4A).

A cumulative risk point was calculated for each patient. The receiver operating characteristic (ROC) curve was used to evaluate the prognostic validity of the model. We used 1 year as the time horizon; that is, patients with OS > 1 year or ≤ 1 year were designated as long and short survival cases, respectively. The area under the ROC curve was larger for the combined risk model than for individual risk factors (Figure 4B), indicating that the model had higher predictive accuracy, which was confirmed by applying the model to the independent set (Figure S7).

## DISCUSSION

Recently, several studies have reported miRNA profiles in GBM, highlighting the role of miRNAs in the progression of this disease [7, 8, 19]. In the present study, 171 miRNAs were found to be differentially expressed between non-tumor brain tissue and GBM specimens with *MGMT* promoter methylation. The majority of these (91.2%) were the same as the 166 miRNAs that were differentially expressed between non-tumor brain tissue and GBM specimens without *MGMT* promoter methylation (Supplementary Data 1). Accordingly, *MGMT* promoter methylation status did not alter the overall miRNA expression profile of GBM. However, few miRNAs with prognostic significance for *MGMT* promoter-methylated GBM showed prognostic significance in unmethylated cases (Supplementary Data 3). Similar findings were obtained using the CGGA dataset (Supplementary Data 4), indicating that most miRNAs exhibit different prognostic values for GBM with and without *MGMT* promoter methylation and highlighting the need for identifying distinct prognostic markers for these two GBM subgroups. A previous study suggested that a multiple miRNA-based risk signature can provide a more statistically robust analysis than individual miRNAs [11]. Accordingly, we developed a five miRNA-based signature with independent prognostic significance for *MGMT* promoter-methylated GBM. While the signature had no prognostic value for patients with an unmethylated *MGMT* promoter, demonstrating that distinct miRNA markers can be used to distinguish between GBM with or without *MGMT* promoter methylation.

Even with equivalent *MGMT* promoter methylation, high- and low-risk patients had distinct prognoses, with the former showing a similar survival to GBM patients with unmethylated *MGMT* promoters. As previously reported, *MGMT* promoter methylation status can also predict responses to alkylating chemotherapy [16]. When we classified *MGMT* promoter-methylated GBM into four subgroups based on the five-miRNA signature and chemotherapy treatment, we found that low-risk patients treated with alkylating agents had the best prognosis; those at high risk who were untreated had the most unfavorable prognosis; and other subgroups had similar survival. High-risk patients with a methylated *MGMT* promoter who were treated with alkylating agents had no survival advantage over low-risk patients who did not receive chemotherapy or those without *MGMT* promoter methylation, suggesting that current chemotherapy strategies are ineffective in these patients. Further studies are needed in order to develop more effective strategies for improving the clinical outcome of high-risk patients. Inhibiting or overexpressing specific miRNAs has therapeutic potential for GBM treatment [20, 21], and the five miRNAs identified here could serve as possible therapeutic targets.

In the combined dataset, a quantitative model was developed consisting of three independent prognostic factors including age, chemotherapy and the five-miRNA signature. As one of the most important prognostic factors for GBM [22], age accounted for a greater proportion of the total risk. Interestingly, chemotherapy and the five-miRNA signature had the same risk points, implying that the clinical benefits of each could be negated. The predictive value of the model was superior to that of individual risk factors, which was validated using an independent set. Therefore, evaluating GBM patients based on this simple model combined with clinicopathologic and molecular information has broad clinical application.

Previous studies have investigated the prognostic roles of the five miRNAs constituting our classifier. In particular, miR-222, -145, and -132 have prognostic significance for GBM patients [23-25]. MiR-222, always along with miR-221, confers a malignant phenotype in GBM [26-28]. These two miRNAs have been shown to regulate *MGMT* expression*,* which added alternative mechanism for MGMT regulation, and thereby increase TMZ sensitivity in GBM [29]. However, in our study miR-222 was found to be associated with a high risk of unfavorable outcome even with alkylating agent treatment. This may be due to the absence of *MGMT* expression resulting from methylation of the promoter. Under these circumstances, miRNA-222 mainly exerts oncogenic potentials, such as cell proliferation and motility, rather than *MGMT* expression regulation. Moreover, only miR-222 showed similar prognostic value for *MGMT* promoter-methylated and -unmethylated patients, suggesting that it is a stable but unfavorable marker [23, 30]. MiR-145 is downregulated in GBM and confers better prognosis [31]. It acts a tumor-suppressive role by targeting genes sex-determining region Y-box 9 and adducin 3, thus mediating invasion and malignant characteristics [32]. MiR-20a has been reported to be expressed at high level in glioma stem cell and enhances the self-renewal of human glioma cells [7, 33]. MiR-132 confers unfavorable prognosis in GBM and is involved in several important functions in other types of cancer, such as angiogenesis, inflammation, and tumorigenesis [25]. MiR-129 has low expression in glioma cells and serves as a tumor suppressor by inhibiting cell growth and invasion [34].

This study was limited by its retrospective nature as well as the small population size of the external validation dataset. For clinical application, a larger independent dataset in a prospective study is required, along with a more extensive investigation of the biological functions of these miRNAs alone and in combination. Considering the fact that miRNAs are stable in serum, several serum miRNAs have been identified as potential biomarkers for noninvasive glioma diagnosis [35, 36], although few studies have investigated their utility in predicting GBM prognosis and treatment response. Serum tests that can detect our five-miRNA signature and its constituent miRNAs would be more convenient and practical in a clinical setting.

In summary, we identified and validated a novel five-miRNA signature with independent prognostic significance for GBM patients with *MGMT* promoter methylation that can be a useful tool for identifying patients who would most benefit from alkylating chemotherapy treatment. Furthermore, a quantitative model combining age, chemotherapy and the five-miRNA signature was established that will allow clinicians to predict patient prognosis and offer more personalized therapeutic regimens.

## MATERIALS AND METHODS

### Patient samples

MiRNA expression data for GBM patients were downloaded from The Cancer Genome Atlas (TCGA) database (http://cancergenome.nih.gov). We excluded patients with an OS time (defined as the interval from the date of diagnosis until death or the last follow-up) of < 30 days, since in these cases, death may have occurred due to factors other than GBM. Thus, a total of 310 primary GBM cases with detailed clinical information and corresponding miRNA data were included in the analysis. GBM cases with *MGMT* promoter methylation (*n* = 150) were selected and randomly divided into training and validation sets (*n* = 75 each) for further analysis. The clinicopathologic features of the two groups are shown in Table 1. In the TCGA combined set, 1 year was set as the time threshold; that is, patients with OS > 1 year or ≤ 1 year were defined as having long and short survival time, respectively. For patients who received alkylating agent treatment, cases with OS > 1 year and ≤ 1 year were defined as chemotherapy responders and non-responders, respectively. Another cohort of 56 GBM samples was selected from the CGGA database for independent validation, which included 19 patients with and 37 without *MGMT* promoter methylation.

### Statistical analysis

All statistical analyses were performed with SPSS software (SPSS Inc., Chicago, IL, USA) and GraphPad Prism 6 (GraphPad Software Inc., La Jolla, CA, USA). Differences in clinical and molecular features between training and validation sets were evaluated with the Student's t or χ^2^ tests. The Student's t test was also used to assess differences in miRNA expression in long *vs*. short survival time and chemotherapy responders *vs*. non-responders.

Prior to analysis, miRNA expression levels were normalized by transformation using the z-score method to enable comparisons between the two datasets. The Student's t test was used to identify miRNAs that are differentially expressed between normal brain tissue and *MGMT* promoter-methylated GBM samples with a threshold FDR < 0.01. The prognostic value of these miRNAs was evaluated by univariate Cox regression analysis in the training set, which identified five miRNAs that were significantly correlated with patient survival time. The five miRNAs formed a risk signature that was determined based on a linear combination of miRNA expression levels weighted with regression coefficients from univariate Cox regression analyses [12, 13]. The prognostic value was estimated using Kaplan-Meier curves and the two-tailed log rank test. Uni- and multivariate Cox regression analyses were carried out to identify independent prognostic factors, which were used to develop a quantitative model for predicting patient outcome. The prediction accuracy of the model was determined from ROC curves. Statistical significance was defined as a two-tailed *P* value < 0.05.

## SUPPLEMENTARY MATERIAL FIGURES AND TABLES




