# Supplementary material for: A five-miRNA signature with prognostic and predictive value for MGMT promoter-methylated glioblastoma patients
Source: Oncotarget. 2015 Jul 22;6(30):29285–95. doi: 10.18632/oncotarget.4978 (PMC4745726; doi:10.18632/oncotarget.4978)
Supplement: Supplementary file 1 [file oncotarget-06-29285-s001.pdf]

A five-miRNA signature with prognostic and predictive value for *MGMT* promoter-methylated glioblastoma patients

Supplementary Material

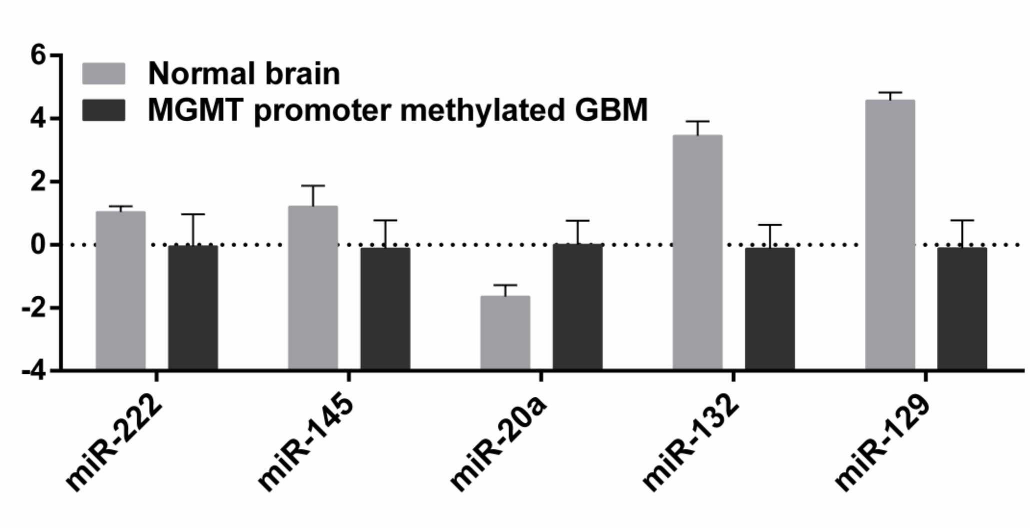

Figure S1. Comparison of the expression levels of five miRNAs comprising the miRNA signature between normal brain tissue and GBM specimens with MGMT promoter methylation. Bars indicate standard deviation.

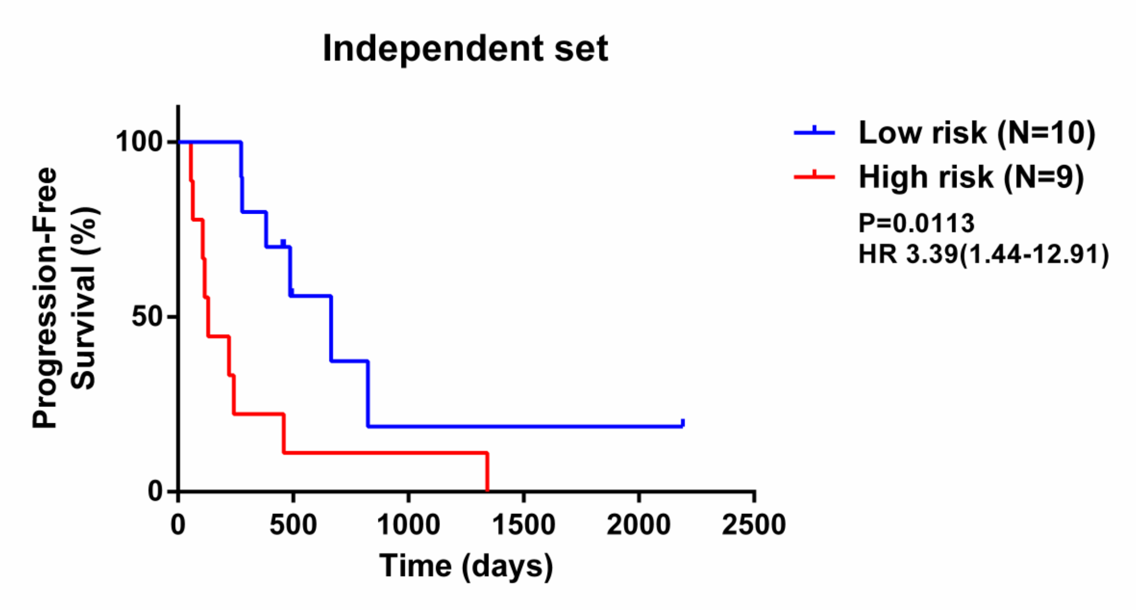

Figure S2. Prognostic value of the five-miRNA signature for PFS using independent set.

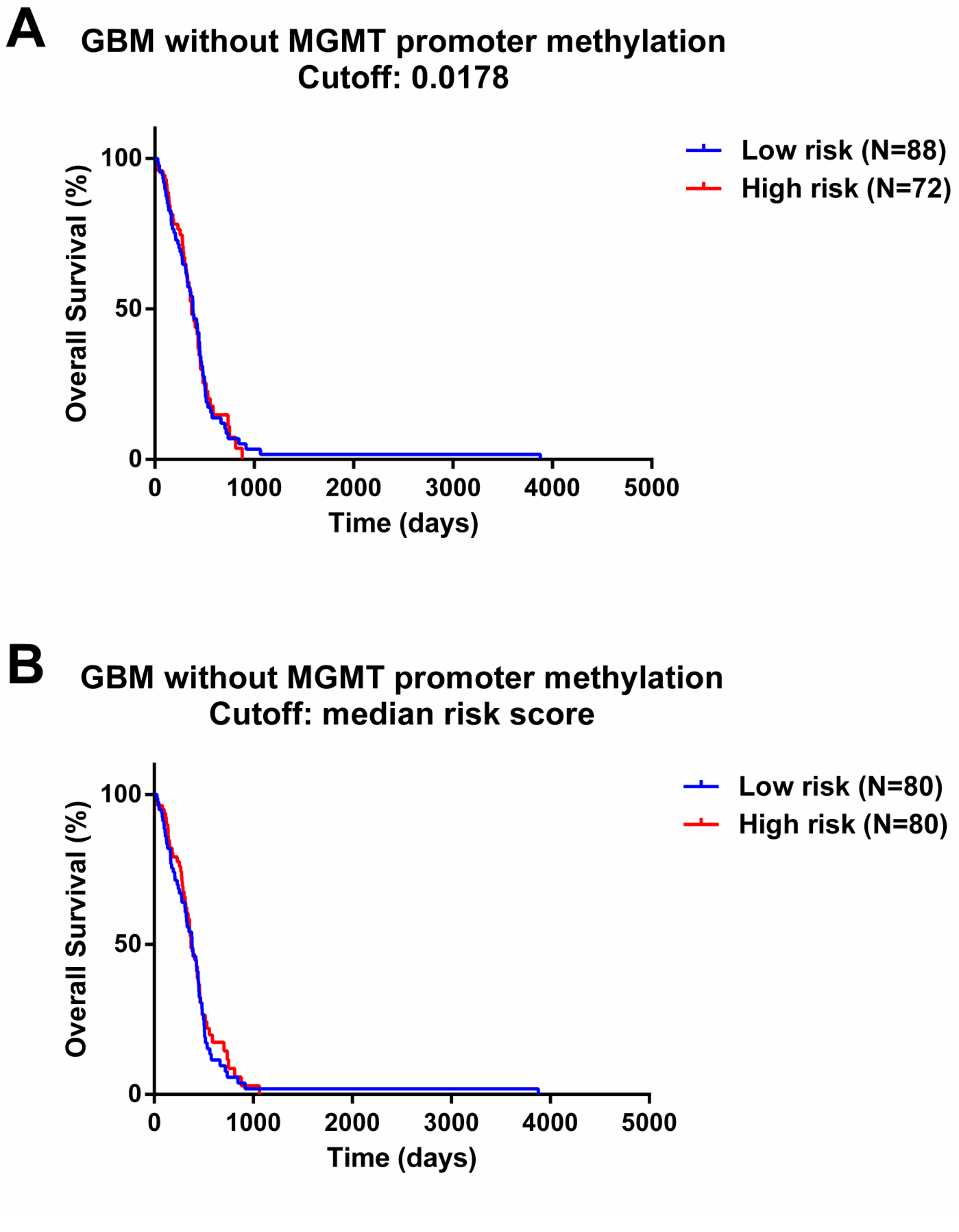

Figure S3. Lack of prognostic value for the five-miRNA signature in GBM patients without *MGMT* promoter methylation using the cutoff value of 0.0178 (A) or the median risk value (B).

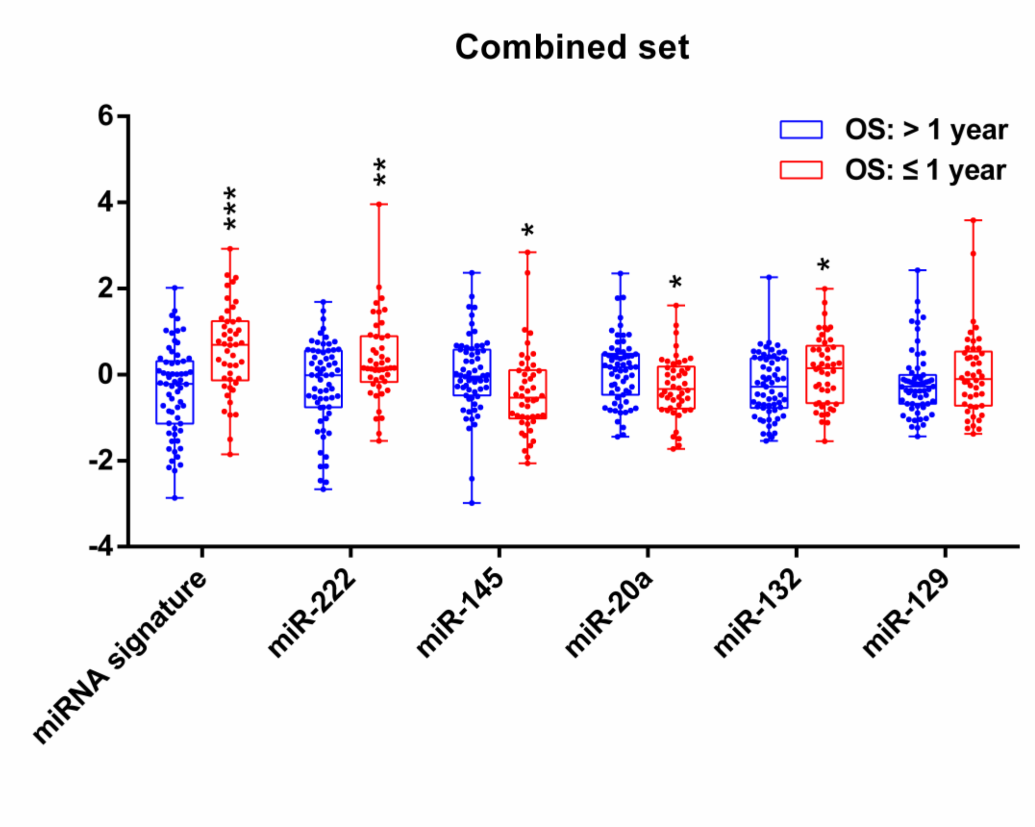

Figure S4. Comparison of the five-miRNA signature and its constituent miRNAs between cases with long and short survival time using a combined set. *P < 0.05, **P < 0.01, ***P < 0.001.

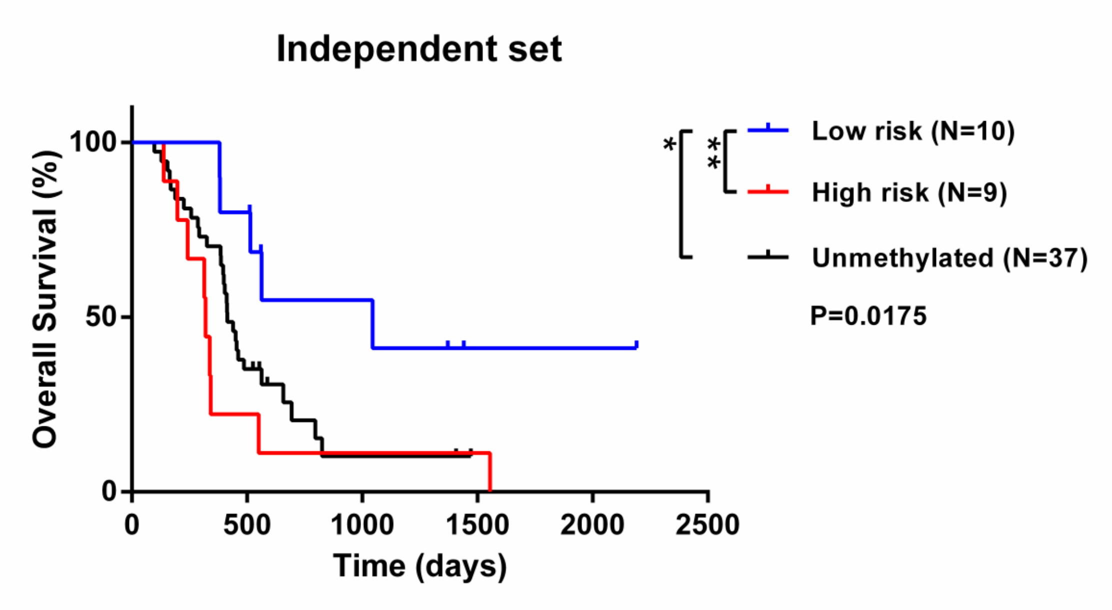

Figure S5. Prognosis of unmethylated GBM samples and high- and low-risk GBM patients with *MGMT* promoter methylation using an independent set. *P < 0.05, **P < 0.01, ***P < 0.001.

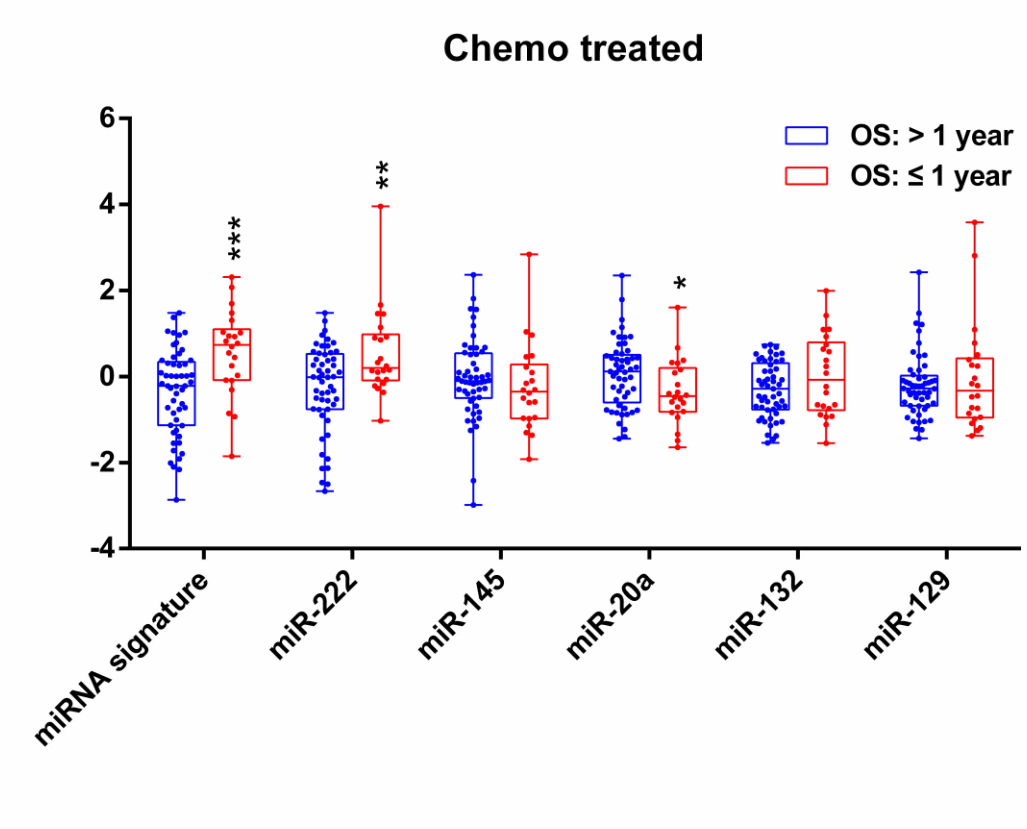

Figure S6. Comparison of the five-miRNA signature and its constituent miRNAs between chemotherapy responders and non-responders. *P < 0.05, **P < 0.01, ***P < 0.001.

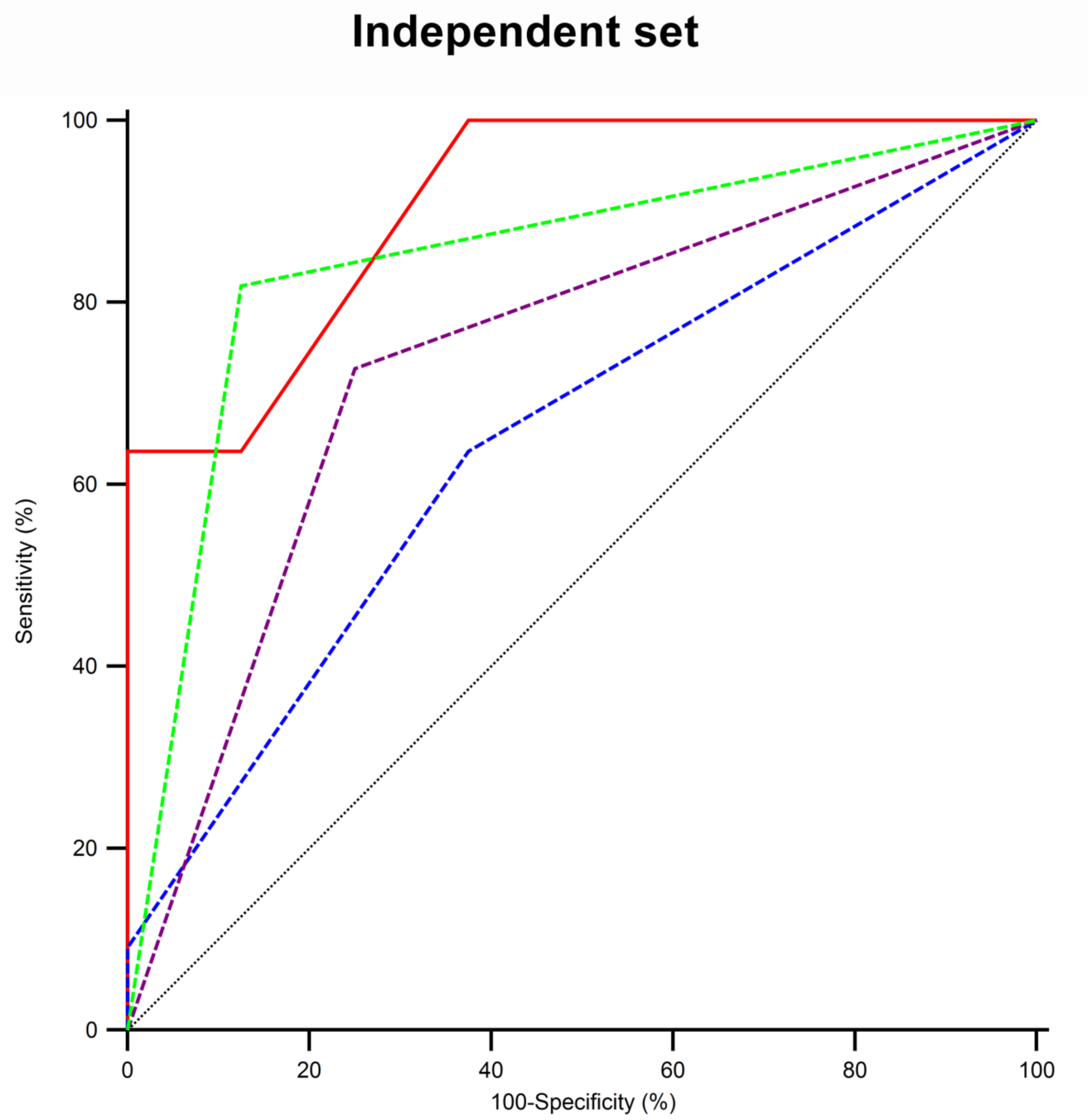

	AUROC	95% CI	P-value
Total points	0.909	0.687 to 0.991	
Age	0.648	0.399 to 0.849	0.0085
Chemotherapy	0.739	0.490 to 0.910	0.0402
5-miRNA signature	0.847	0.610 to 0.968	0.4524

Figure S7. Sensitivity and specificity of the risk model for predicting 1-year survival using the independent set.

Table S1. Univariate Cox regression of miRNAs for survival in training set.

**Variable**	**P-value**	**HR**	**95%CI**
hsa-miR-222	0.0112	1.6024	1.1129-2.3072
hsa-miR-145	0.0132	0.5809	0.3780-0.8925
hsa-miR-20a	0.0202	0.6175	0.4112-0.9274
hsa-miR-132	0.0218	1.6954	1.0797-2.6623
hsa-miR-129	0.0456	1.3948	1.0066-1.9328

Table S2. Cox hazard regression analysis of clinicopathologic factors and the five-miRNA signature for survival in the independent set

**Variable**	**Univariate Cox**		**Multivariate Cox**	
	P-value	HR	P-value	HR
**Age**				
(Per 20 years)	0.3845	1.4990		
**Gender**				
(Female vs. Male)	0.5433	0.7195		
**KPS**				
(≥80 vs. <80)	0.4431	0.5296		
**IDH1 mutation**				
(Mutation vs. Wild type)	0.0469	0.1137	0.0305	0.0753
**Radiotherapy**				
(Treated vs. Untreated)	0.0530	0.2504		
**Chemotherapy**				
(Treated vs. Untreated)	0.0937	0.3798		
**Five-miRNA signature**				
(High vs. Low risk)	**0.0082**	**4.5896**	**0.0090**	**6.4662**
